# Limited by our limitations

**DOI:** 10.1007/s40037-019-00530-x

**Published:** 2019-07-25

**Authors:** Paula T. Ross, Nikki L. Bibler Zaidi

**Affiliations:** Medical School, University of Michigan, Ann Arbor, MI USA

**Keywords:** Research, Limitations

## Abstract

Study limitations represent weaknesses within a research design that may influence outcomes and conclusions of the research. Researchers have an obligation to the academic community to present complete and honest limitations of a presented study. Too often, authors use generic descriptions to describe study limitations. Including redundant or irrelevant limitations is an ineffective use of the already limited word count. A meaningful presentation of study limitations should describe the potential limitation, explain the implication of the limitation, provide possible alternative approaches, and describe steps taken to mitigate the limitation. This includes placing research findings within their proper context to ensure readers do not overemphasize or minimize findings. A more complete presentation will enrich the readers’ understanding of the study’s limitations and support future investigation.

## Introduction

Regardless of the format scholarship assumes, from qualitative research to clinical trials, all studies have limitations. Limitations represent weaknesses within the study that may influence outcomes and conclusions of the research. The goal of presenting limitations is to provide meaningful information to the reader; however, too often, limitations in medical education articles are overlooked or reduced to simplistic and minimally relevant themes (e.g., single institution study, use of self-reported data, or small sample size) [1]. This issue is prominent in other fields of inquiry in medicine as well. For example, despite the clinical implications, medical studies often fail to discuss how limitations could have affected the study findings and interpretations [2]. Further, observational research often fails to remind readers of the fundamental limitation inherent in the study design, which is the inability to attribute causation [3]. By reporting generic limitations or omitting them altogether, researchers miss opportunities to fully communicate the relevance of their work, illustrate how their work advances a larger field under study, and suggest potential areas for further investigation.

## Goals of presenting limitations

Medical education scholarship should provide empirical evidence that deepens our knowledge and understanding of education [4, 5], informs educational practice and process, [6, 7] and serves as a forum for educating other researchers [8]. Providing study limitations is indeed an important part of this scholarly process. Without them, research consumers are pressed to fully grasp the potential exclusion areas or other biases that may affect the results and conclusions provided [9]. Study limitations should leave the reader thinking about opportunities to engage in prospective improvements [9–11] by presenting gaps in the current research and extant literature, thereby cultivating other researchers’ curiosity and interest in expanding the line of scholarly inquiry [9].

Presenting study limitations is also an ethical element of scientific inquiry [12]. It ensures transparency of both the research and the researchers [10, 13, 14], as well as provides transferability [15] and reproducibility of methods. Presenting limitations also supports proper interpretation and validity of the findings [16]. A study’s limitations should place research findings within their proper context to ensure readers are fully able to discern the credibility of a study’s conclusion, and can generalize findings appropriately [16].

## Why some authors may fail to present limitations

As Price and Murnan [8] note, there may be overriding reasons why researchers do not sufficiently report the limitations of their study. For example, authors may not fully understand the importance and implications of their study’s limitations or assume that not discussing them may increase the likelihood of publication. Word limits imposed by journals may also prevent authors from providing thorough descriptions of their study’s limitations [17]. Still another possible reason for excluding limitations is a diffusion of responsibility in which some authors may incorrectly assume that the journal editor is responsible for identifying limitations. Regardless of reason or intent, researchers have an obligation to the academic community to present complete and honest study limitations.

## A guide to presenting limitations

The presentation of limitations should describe the potential limitations, explain the implication of the limitations, provide possible alternative approaches, and describe steps taken to mitigate the limitations. Too often, authors only list the potential limitations, without including these other important elements.

### Describe the limitations

When describing limitations authors should identify the limitation type to clearly introduce the limitation and specify the origin of the limitation. This helps to ensure readers are able to interpret and generalize findings appropriately. Here we outline various limitation types that can occur at different stages of the research process.

#### Study design

Some study limitations originate from conscious choices made by the researcher (also known as delimitations) to narrow the scope of the study [1, 8, 18]. For example, the researcher may have designed the study for a particular age group, sex, race, ethnicity, geographically defined region, or some other attribute that would limit to whom the findings can be generalized. Such delimitations involve conscious exclusionary and inclusionary decisions made during the development of the study plan, which may represent a systematic bias intentionally introduced into the study design or instrument by the researcher [8]. The clear description and delineation of delimitations and limitations will assist editors and reviewers in understanding any methodological issues.

#### Data collection

Study limitations can also be introduced during data collection. An unintentional consequence of human subjects research is the potential of the researcher to influence how participants respond to their questions. Even when appropriate methods for sampling have been employed, some studies remain limited by the use of data collected only from participants who decided to enrol in the study (self-selection bias) [11, 19]. In some cases, participants may provide biased input by responding to questions they believe are favourable to the researcher rather than their authentic response (social desirability bias) [20–22]. Participants may influence the data collected by changing their behaviour when they are knowingly being observed (Hawthorne effect) [23]. Researchers—in their role as an observer—may also bias the data they collect by allowing a first impression of the participant to be influenced by a single characteristic or impression of another characteristic either unfavourably (horns effect) or favourably (halo effort) [24].

#### Data analysis

Study limitations may arise as a consequence of the type of statistical analysis performed. Some studies may not follow the basic tenets of inferential statistical analyses when they use convenience sampling (i.e. non-probability sampling) rather than employing probability sampling from a target population [19]. Another limitation that can arise during statistical analyses occurs when studies employ unplanned post-hoc data analyses that were not specified before the initial analysis [25]. Unplanned post-hoc analysis may lead to statistical relationships that suggest associations but are no more than coincidental findings [23]. Therefore, when unplanned post-hoc analyses are conducted, this should be clearly stated to allow the reader to make proper interpretation and conclusions—especially when only a subset of the original sample is investigated [23].

#### Study results

The limitations of any research study will be rooted in the validity of its results—specifically threats to internal or external validity [8]. Internal validity refers to reliability or accuracy of the study results [26], while external validity pertains to the generalizability of results from the study’s sample to the larger, target population [8].

Examples of threats to internal validity include: effects of events external to the study (history), changes in participants due to time instead of the studied effect (maturation), systematic reduction in participants related to a feature of the study (attrition), changes in participant responses due to repeatedly measuring participants (testing effect), modifications to the instrument (instrumentality) and selecting participants based on extreme scores that will regress towards the mean in repeat tests (regression to the mean) [27].

Threats to external validity include factors that might inhibit generalizability of results from the study’s sample to the larger, target population [8, 27]. External validity is challenged when results from a study cannot be generalized to its larger population or to similar populations in terms of the context, setting, participants and time [18]. Therefore, limitations should be made transparent in the results to inform research consumers of any known or potentially hidden biases that may have affected the study and prevent generalization beyond the study parameters.

### Explain the implication(s) of each limitation

Authors should include the potential impact of the limitations (e.g., likelihood, magnitude) [13] as well as address specific validity implications of the results and subsequent conclusions [16, 28]. For example, self-reported data may lead to inaccuracies (e.g. due to social desirability bias) which threatens internal validity [19]. Even a researcher’s inappropriate attribution to a characteristic or outcome (e.g., stereotyping) can overemphasize (either positively or negatively) unrelated characteristics or outcomes (halo or horns effect) and impact the internal validity [24]. Participants’ awareness that they are part of a research study can also influence outcomes (Hawthorne effect) and limit external validity of findings [23]. External validity may also be threatened should the respondents’ propensity for participation be correlated with the substantive topic of study, as data will be biased and not represent the population of interest (self-selection bias) [29]. Having this explanation helps readers interpret the results and generalize the applicability of the results for their own setting.

### Provide potential alternative approaches and explanations

Often, researchers use other studies’ limitations as the first step in formulating new research questions and shaping the next phase of research. Therefore, it is important for readers to understand why potential alternative approaches (e.g. approaches taken by others exploring similar topics) were not taken. In addition to alternative approaches, authors can also present alternative explanations for their own study’s findings [13]. This information is valuable coming from the researcher because of the direct, relevant experience and insight gained as they conducted the study. The presentation of alternative approaches represents a major contribution to the scholarly community.

### Describe steps taken to minimize each limitation

No research design is perfect and free from explicit and implicit biases; however various methods can be employed to minimize the impact of study limitations. Some suggested steps to mitigate or minimize the limitations mentioned above include using neutral questions, randomized response technique, force choice items, or self-administered questionnaires to reduce respondents’ discomfort when answering sensitive questions (social desirability bias) [21]; using unobtrusive data collection measures (e.g., use of secondary data) that do not require the researcher to be present (Hawthorne effect) [11, 30]; using standardized rubrics and objective assessment forms with clearly defined scoring instructions to minimize researcher bias, or making rater adjustments to assessment scores to account for rater tendencies (halo or horns effect) [24]; or using existing data or control groups (self-selection bias) [11, 30]. When appropriate, researchers should provide sufficient evidence that demonstrates the steps taken to mitigate limitations as part of their study design [13].

## Conclusion

In conclusion, authors may be limiting the impact of their research by neglecting or providing abbreviated and generic limitations. We present several examples of limitations to consider; however, this should not be considered an exhaustive list nor should these examples be added to the growing list of generic and overused limitations. Instead, careful thought should go into presenting limitations after research has concluded and the major findings have been described. Limitations help focus the reader on key findings, therefore it is important to only address the most salient limitations of the study [17, 28] related to the specific research problem, not general limitations of most studies [1]. It is important not to minimize the limitations of study design or results. Rather, results, including their limitations, must help readers draw connections between current research and the extant literature.

The quality and rigor of our research is largely defined by our limitations [31]. In fact, one of the top reasons reviewers report recommending acceptance of medical education research manuscripts involves limitations—specifically how the study’s interpretation accounts for its limitations [32]. Therefore, it is not only best for authors to acknowledge their study’s limitations rather than to have them identified by an editor or reviewer, but proper framing and presentation of limitations can actually increase the likelihood of acceptance. Perhaps, these issues could be ameliorated if academic and research organizations adopted policies and/or expectations to guide authors in proper description of limitations.
